# In This Issue

**Published:** 2006

## CHARTING A PATH BETWEEN RESEARCH AND PRACTICE IN ALCOHOLISM TREATMENT

Researchers and clinicians approach alcoholism treatment from two very different perspectives, a fact that may make it difficult to link these two disciplines. Researchers, on the one hand, are concerned with using standardized techniques and proper data collection. Therapists, on the other hand, tend to focus on practical matters and on making the most of limited resources. The value of applying research findings to practice and of using clinical insights to guide research makes it important to connect the research and practice worlds. Drs. Dennis McCarty and Eldon Edmundson, Jr., and Mr. Tim Hartnett describe the journey between research and practice and the factors that influence successful navigation along this path. The authors provide examples of specific pharmaceutical and behavioral interventions to illustrate how research-based treatment approaches can be implemented in clinical settings. (pp. 5–10)

## TRANSLATING RESEARCH FINDINGS INTO PRACTICE: EXAMPLE OF TREATMENT SERVICES FOR ADOLESCENTS IN MANAGED CARE

Many researchers are investigating how to improve the effectiveness of alcoholism treatment for various population subgroups. The diverse interests and concerns of a variety of stakeholders (e.g., health plan administrators, program administrators, mental health and primary care providers, and patients), however, often are not adequately represented in the development of these studies. This disconnect tends to inhibit the integration of study findings into real-world treatment settings. Ms. Stacy Sterling and Dr. Constance Weisner present a novel research–practice integration model designed to facilitate the transfer of research findings into clinical practice and to incorporate stakeholder concerns into the research process. Researchers successfully applied this model to an adolescent alcohol and other drug treatment program in a managed health care plan. (pp. 11–18)

## PERFORMANCE MEASURES FOR ALCOHOL AND OTHER DRUG SERVICES

Performance measures—which evaluate the extent to which health care practitioners’ actions conform to practice guidelines, medical review criteria, or standards of quality—can improve access to treatment services and the quality of those services for people with alcohol and other drug problems. Drs. Deborah W. Garnick, Constance M. Horgan, and Mady Chalk describe three important variables that figure into the development and use of performance measures: the types of quality measures, how they fit within the continuum of care, and the types of data from which these measures can be derived. The authors highlight the widely used set of performance measures developed by the Washington Circle, describing the development, testing, implementation, and adoption of these measures. (pp. 19–26)

## ECONOMIC EVALUATION OF ALCOHOLISM TREATMENT

Current concern over rising health care costs means that economic considerations influence treatment decisions in all areas of medicine, including alcoholism treatment. Studies determining the cost and cost-effectiveness of different treatment approaches can help ensure that people with alcohol-related problems receive appropriate care. Drs. Jeremy W. Bray and Gary A. Zarkin describe several methods of economic analysis that investigators employ for such studies, including cost analyses, cost-effectiveness analyses, and cost–benefit analyses, and explain the type of research question each method best addresses as well as the strengths and weaknesses of each method. This area of health services research will continue to evolve as new alcoholism treatment approaches are developed and the economic analytic methods used to evaluate them are refined. (pp. 27–33)

## ANALYZING THE COSTS AND BENEFITS OF BRIEF INTERVENTION

The Trial for Early Alcohol Treatment—Project TrEAT—is one of the few brief interventions that has been analyzed in terms of its cost-effectiveness. Project TrEAT was a randomized controlled trial of screening and brief intervention in primary care clinics. It consisted of two 15-minute sessions with a physician 4 weeks apart and a followup call from a clinic nurse 2 weeks after each physician session. As described by Mr. Marlon P. Mundt, researchers analyzed the cost-effectiveness of the Project TrEAT interventions from two perspectives—that of the medical care provider and that of society at large—and included the calculation of a benefit–cost ratio for each perspective. The analysis from the medical care provider perspective was limited to clinic and hospital costs; it contrasted the benefits that directly reduced medical expenditures with the costs to providers. The analysis from the societal perspective took all costs and benefits of the intervention into account. Overall, this economic analysis supports the cost-effectiveness of the brief intervention used in Project TrEAT: the benefits—a reduction in drinking levels among high-risk drinkers and a corresponding reduction in medical and societal costs— outweigh the costs of the intervention. (pp. 34–36)

## COMPUTER-BASED TOOLS FOR DIAGNOSIS AND TREATMENT OF ALCOHOL PROBLEMS

Computers can play an important part in increasing the cost-effectiveness of alcoholism treatment and enhancing treatment accessibility. According to Dr. Reid K. Hester and Mr. Joseph H. Miller, computer-based approaches can provide immediate, personalized feedback to the client; minimize bias that could arise in the client–provider relationship; and store information for later analysis and followup. Clinicians can use computer programs when assessing alcohol problems and intervening with clients identified as having alcohol problems; computer programs also can assist in increasing the patient’s motivation to change or reducing harm associated with drinking. Despite studies showing the validity and effectiveness of computer-based assessments and interventions, many providers and treatment programs remain reluctant to use them with their clients. (pp. 36–40)

## COURT-MANDATED TREATMENT FOR CONVICTED DRINKING DRIVERS

Court-mandated treatment for people convicted of driving under the influence of alcohol (DUI) requires offenders to participate in treatment for their substance abuse problems or face legal consequences. Mandated treatment takes many forms, and research has found some types to be more effective than others, explain Drs. Patricia L. Dill and Elisabeth Wells-Parker. The authors also discuss DUI events as opportunities for intervention; screening and assessment/referral for mandated clients; brief interventions for offenders outside of mandated treatment; and the cost-effectiveness of mandated treatment. Areas for future research include the changing DUI population, impaired driving and multidrug use, and new technologies for monitoring DUI offenders. (pp. 41–48)

## UNEQUAL TREATMENT: RACIAL AND ETHNIC DISPARITIES IN ALCOHOLISM TREATMENT SERVICES

The rates, severity, and consequences of clinically significant alcohol problems are higher in some minority populations in the United States than among Whites, studies show. As Drs. Laura Schmidt, Thomas Greenfield, and Nina Mulia report, however, studies evaluating access to and utilization of alcoholism treatment for different racial and ethnic groups have produced ambiguous results. It is clear that disparities exist in the quality and appropriateness of the care received by different populations. For example, minority clients may have to wait longer before they can begin treatment, they do not stay in treatment as long, and they are less satisfied with the treatment they receive. Whether treatments targeted to different ethnic groups can improve treatment effectiveness is still a matter of debate, and like many other aspects of racial and ethnic disparities in alcoholism treatment, this requires further study. (pp. 49–54)

## GENDER AND USE OF SUBSTANCE ABUSE TREATMENT SERVICES

Men traditionally have been the focus of studies on substance abuse treatment. Research efforts in recent decades, however, have helped to close the gender gap. Likewise, many treatment programs have begun to pay greater attention to female patients and their special needs. Today, treatment programs are beginning to offer gender-specific services and ancillary assistance such as child care and parenting groups, which make it easier for women to both enter and continue treatment. Dr. Carla A. Green reviews the current research addressing gender differences in treatment-seeking, access to care, retention in care, and treatment outcomes. As Dr. Green explains, women are more likely than men to face multiple barriers in accessing treatment and are less likely to seek treatment. Women also tend to seek treatment for their alcohol-related problems in mental health or primary care settings rather than in specialized treatment programs, and this could contribute to poorer treatment outcomes. When gender differences in treatment outcomes are considered, however, women tend to fare better than men. Limited research suggests that gender-specific treatment is no more effective than mixed-gender treatment, though some women may only seek treatment in women-targeted programs. (pp. 55–62)

## WELFARE REFORM AND SUBSTANCE ABUSE TREATMENT FOR WELFARE RECIPIENTS

The 1996 welfare reform law set time limits on benefits and required recipients to work, including recipients with substance use disorders. The welfare reform law’s requirements may have important implications for low-income people with substance use disorders and the programs that serve them. Drs. Jon Morgenstern and Kimberly A. Blanchard report on the prevalence of substance use and substance use disorders among recipients of Temporary Assistance for Needy Families benefits. They address the extent to which people with substance use disorders and co-occurring problems have trouble getting and keeping jobs; whether welfare offices are good places to screen and identify people for substance abuse problems and refer them to substance abuse treatment; and the types of services people with substance use disorders need in order to be self-sufficient. The authors also offer suggestions for how these findings can inform policy and future research. (pp. 63–67)

